# Lychee Seed Saponins Improve Cognitive Function and Prevent Neuronal Injury via Inhibiting Neuronal Apoptosis in a Rat Model of Alzheimer’s Disease

**DOI:** 10.3390/nu9020105

**Published:** 2017-02-04

**Authors:** Xiuling Wang, Jianming Wu, Chonglin Yu, Yong Tang, Jian Liu, Haixia Chen, Bingjin Jin, Qibing Mei, Shousong Cao, Dalian Qin

**Affiliations:** 1 Laboratory of Chinese Materia Medica, Department of Pharmacology, School of Pharmacy, Southwest Medical University, Luzhou 646000, Sichuan, China; wangxiulingbest@gmail.com (X.W.); jianmingwu@swmu.edu.cn (J.W.); tangy1989@yeah.net (Y.T.); belkn@sohu.com (J.L.); dna0805001@163.com (H.C.); jinbingjinyy@126.com (B.J.); qbmei@fmmu.edu.cn (Q.M.); 2 Department of Human Anatomy, School of Preclinical Medicine, Southwest Medical University, Luzhou 646000, Sichuan, China; chonglinyu@swmu.edu.cn; 3 Laboratory of Cancer Pharmacology, Department of Pharmacology, School of Pharmacy, Southwest Medical University, Luzhou 646000, Sichuan, China

**Keywords:** lychee seed saponins, Alzheimer’s disease, rat model of Alzheimer’s disease, cognitive function, neuronal injury, apoptosis

## Abstract

Lychee seed is a traditional Chinese medicine and possesses many activities, including hypoglycemia, liver protection, antioxidation, antivirus, and antitumor. However, its effect on neuroprotection is still unclear. The present study investigated the effects of lychee seed saponins (LSS) on neuroprotection and associated mechanisms. We established a rat model of Alzheimer’s disease (AD) by injecting Aβ_25–35_ into the lateral ventricle of rats and evaluated the effect of LSS on spatial learning and memory ability via the Morris water maze. Neuronal apoptosis was analyzed by hematoxylin and eosin stain and terminal deoxynucleotidyl transferase (Tdt)-mediated dUTP nick-end labeling analysis, and mRNA expression of caspase-3 and protein expressions of Bax and Bcl-2 by reverse transcription-polymerase chain reaction (RT-PCR) and Western blotting, respectively. The results showed that LSS remarkably improved cognitive function and alleviated neuronal injury by inhibiting apoptosis in the hippocampus of AD rats. Furthermore, the mRNA expression of caspase-3 and the protein expression of Bax were downregulated, while the protein expression of Bcl-2 and the ratio of Bcl-2/Bax were increased by LSS. We demonstrate that LSS significantly improves cognitive function and prevent neuronal injury in the AD rats via regulation of the apoptosis pathway. Therefore, LSS may be developed as a nutritional supplement and sold as a drug for AD prevention and/or treatment.

## 1. Introduction

As a consequence of global aging, Alzheimer’s disease (AD) is rapidly becoming a very common progressive neurodegenerative disorder that has the highest incidence in the world. Accumulating evidence shows that the major pathological causes of AD are the abnormal accumulation of senile plaques (SPs) containing amyloid-β protein (Aβ) and neurofibrillary tangles (NFTs) consisting of hyperphosphorylated tau in the brain [1,2]. Although the exact molecular mechanism for the neurodegeneration in AD is still unknown, current knowledge from the basic and clinical studies in the pathogenesis of AD indicates that neuronal apoptosis plays the key role in neuronal injury and loss in AD [3,4,5]. Neuronal apoptosis is influenced by various factors, such as Bcl-2, Bax, caspases, Aβ, tumor necrosis factor-α (TNF-α), amyloid precursor protein intracellular *C*-terminal domain, reactive oxygen species (ROS), perturbation of enzymes, and the possible causes of AD [6]. However, a comprehensive understanding of this process is still lacking due to the complexity of the molecular mechanisms involved in the various triggering events and the signaling cascades leading to neuronal apoptosis. The underlying mechanism involved in neuronal apoptosis in AD may be associated with multiple gene expression, such as Bcl-2-associated death promoter (BAD), inhibitor of apoptosis protein (IAP), cytochrome c (CytC), TNF receptor 1 (TNF-R1), and so on [7]. Studies have shown that pro-apoptotic kinases may be the link between Aβ and tau anomalies, and three proapoptotic kinases, including double-stranded RNA kinase (PKR), glycogen synthase kinase-3β, and *C*-Jun terminal kinase (JNK), have been implicated in AD physiopathology [8]. Overexpression of miRNA-146a (a brain-enriched miRNA and upregulated in AD patients) led to reduction of Akt activation and induction of proapoptotic caspase-3 to induce cell apoptosis via inhibition of lipoprotein-related protein 2 (LRP2) [9]. Caspases are crucial mediators of apoptosis and caspase-3 is essential for normal brain development and is important, or essential, for certain processes associated with apoptosis [10].

Aβ, one of the main toxic peptides and a hallmark of AD, is generated from amyloid-β protein precursor (APP) processing by β- and γ-secretases through the amyloid cascade pathway [11]. Aβ itself can inhibit insulin signaling pathways leading to the dysfunction of downstream pathways (PI_3_-K/Akt, MAPK, and Wnt) [12,13,14] and a variety of characteristic pathological changes in AD, including accumulation of Aβ, tau protein hyperphosphorylation, and neuronal apoptosis [15,16]. Studies have demonstrated that insulin signal pathway affects neuronal apoptosis in diabetic mice through inhibiting pro-apoptosis protein families such as BAD and glycogen synthase kinase 3β (GSK-3β) [17,18,19]. BAD can inhibit anti-apoptosis Bcl-2 family proteins, resulting in neuronal apoptosis [20].

Currently, only five drugs have been approved for the treatment of AD; among them, four are acetylcholinesterase inhibitors (donepezil, tacrine, rivastigmine, and galantamine) and one NMDA receptor antagonist (memantine). Donepezil (Aricept), a centrally acting reversible acetylcholinesterase inhibitor, is the first choice for AD treatment recommended by the National Institute for Health and Care Excellence (NICE) guidance. Donepezil, like all other AD drugs, only improves cognition and behavior of the patients with AD, but cannot slow the progression or cure the disease [21]. In addition, donepezil has many common side effects including loss of appetite, gastrointestinal upset, diarrhea, vomiting, difficulty sleeping, and muscle cramping [22]. The clinical trials of AD drugs had a very high failure rate and most phase II trials with a positive outcome do not succeed in phase III due to lack of therapeutic efficacy or serious adverse effects [23]. No new drug has been approved for the treatment of AD since 2003. Therefore, discovery and development of more effective and/or less toxic novel anti-AD drugs or nutrient supplements are urgently needed.

Natural products and medicinal herbs are important source of protective compounds against AD and the use of drug substances derived from natural sources has a long tradition in medicine [24,25]. Recently, active ingredients from natural products and medicinal herbs for treatment of AD have attracted substantial attention. For example, some natural polyphenols and flavonoids have been revealed to have a variety of biological actions and neuroprotective effects that constitute the basis for AD treatment [26,27]. However, poor bioavailability and low clinical efficacy are the major obstacles [11]. Lychee seed (Semen Litchi, Litchi nut) is a famous traditional Chinese medicine named “Li-zhi-he” in Chinese and was recorded by the Bencao Gangmu (Compendium of Materia Medica). Some components of lychee seed possess multiple activities such as modulation of blood glucose by decreasing plasma glucose, improving insulin resistance (IR), and sensitivity [28,29], lowering blood lipids [30], preventing liver injury [31], antioxidation [32], antivirus [33], antitumor [34] and so on. The chemical components of lychee seed comprise saponins, volatile oils, organic acids, fatty acids, amino acids, flavonoids, and sugar [31]. Lychee seed aqueous extracts improved the learning and memory of mice [35].

Study has determined the contents of anti-diabetic saponins from lychee seed [36]. Our preliminary studies have found that the extracts from lychee seed significantly decreased the formation of advanced glycation end products (AGEs), Aβ, and tau protein in the brain of tested rats, and relieved the cognitive dysfunction in T2DM rats by improving insulin resistance In order to study the effect of lychee seed saponins (LSS) on the brain function of the rats with AD, we isolated and extracted the total saponins from lychee seeds and analyzed the chemical profile of LSS by an ultra-high performance liquid chromatography system with a SPD UV/VIS detector (UHPLC-SPD, Shimadzu Corp., Nakagyo-ku, Kyoto, Japan). Meanwhile, we established a rat model of AD by injecting Aβ_25–35_ into the lateral ventricle of rats and investigated the spatial learning and memory ability of the rats with the Morris water maze. We also studied neuropathology and apoptosis of the neurons of rats by hematoxylin and eosin (H&E) stain and terminal deoxynucleotidyl transferase dUTP nick end labeling (TUNEL) analysis. Furthermore, to explore the possible mechanisms associated with the effects of LSS on brain function and neuronal injury in vivo, we investigated the mRNA expression of caspase-3 in the brain tissues of rats by reverse transcription-polymerase chain reaction (RT-PCR) and the protein expressions of Bax and Bcl-2 in the rat adrenal pheochromocytoma 12 (PC12) cells by Western blotting in vitro.

## 2. Materials and Methods

### 2.1. General Information

Donepezil hydrochloride table (Lot: 110822A) was purchased from Eisai China Inc. (Suzhou, Jiangsu, China) and prepared to a mixed suspension at the concentration of 0.1 mg/mL. Aβ_25–35_ (Lot: Y-0044) was purchased from BIOSS Biotech Limited Company (Beijing, China). Streptavidin-biotin complex (SABC) and TUNEL apoptosis detection kits were purchased from BOSTER Biotech Limited Company (Wuhan, Hubei, China). An RT-PCR kit was purchased from TransGen Biotech Limited Company (Beijing, China). Anti-Bcl-2, anti-Bax, and anti-β-actin antibodies were purchased from Bio-Rad Laboratories, Inc. (Berkeley, CA, USA). The MT-2000 Morris water maze was purchased from Chengdu Taimeng Technology Co., Ltd. (Chengdu, Sichuan, China). A ZH-LAN-STAR C type brain stereotaxic apparatus was purchased from Huaibei Zhenhua Biological Equipment Co., Ltd. (Huaibei, Anhui, China). An inverted microscope S70 was purchased from Leica DMIRB (Lognes, France). A ChemiDocXRS gel imaging and analysis system was purchased from Bio-Rad Laboratories, Inc. (Berkeley, CA, USA). An SP-9000 immunohistochemistry kit was purchased from Beijing ZSGB Biological Engineering Co., Ltd. (Beijing, China). A TC-512 PCR amplification instrument was purchased from Techen (London, UK).

### 2.2. Plant Material and Extraction

Lychee seeds were purchased from the local market in Luzhou (Sichuan, China) and were authenticated by Professor Can Tang of the Department of Chinese Materia Medica, School of Pharmacy, Southwest Medical University (Luzhou, Sichuan, China). The air-dried lychee seeds (1000 g) were ground and soaked with 70% ethanol (1000 mL) overnight, then extracted by percolation with 70% ethanol (8000 mL), the percolate speed is 5 mL/min/kg. The solvents (8370 mL) were evaporated under vacuum and diluted to 5000 mL with distilled water. The LSS in the percolation was absorbed by D101 macroporous resins with speed of 6 mL/min, then the LSS was collected by elution with 70% ethanol (800 mL) and concentrated to dryness using a rotary vacuum evaporator, yielded 31.75 g dried extract.

### 2.3. Analysis of the Chemical Profile of LSS by UHPLC-SPD Chromatogram

The chemical profile of LSS was performed at 210 nm by a Shimadzu LCMS-8040 UHPLC system (Shimadzu Corp.) comprised of two LC-30AD pumps, a SIL-30AC autosampler with a CTO-30AC column oven, a DGU-20A5 degasser, and a Shimadzu CBM-20A system controller. Chromatographic analyses were achieved at 45 °C with an InertSustain C18 column (GL Science, 2.0 μM particle size, 50 mm × 2.1 mm, Shimadzu Corp.), using water-formic acid (100:0.1, *v*/*v*) and methanol as the mobile phase A and phase B, respectively. The mobile phase was delivered at a rate of 0.25 mL/min. The injection volume was 1 μL. For the gradient separation, the gradient program was processed as follows: 15%–15% B at 0–2.0 min, 15%–80% B at 2.0–5.0 min, 80%–80% B at 5.0–7.0 min, 80%–15% B at 7.0–7.5 min. The data analysis was performed using LabSolutions software (Version 5.75, Shimadzu Corp.).

### 2.4. Experimental Animals

Six-month-old specific-pathogen-free (SPF) grade male Sprague Dawley (SD) rats (body weighing 180–220 g) were purchased from the Experimental Animal Centers, Sichuan Provincial Academy of Medical Sciences (Chengdu, Sichuan, China, Certificate No. SCXK201302). The rats were housed in normal plastic cages with free access to water and diet at a constant room temperature (~25 °C) in a 12-h light/12-h dark cycle. All animal experiments were performed strictly in accordance with institutional guidelines and were approved (Permit No. 250114) by the Committee on Use and Care of Animals of Southwest Medical University (Luzhou, Sichuan, China).

### 2.5. Morris Water Maze Test

The spatial learning and memory ability was investigated using the Morris water maze. The general testing procedure was described previously [37]. Briefly, the test was conducted in a round white pool (94 cm in diameter and 31 cm deep) filled with water (30 cm depth) with 3000 mL skim milk at a temperature of approximately 25 °C. The escape platform was a 25-cm^2^ Plexiglas square, placed in the center of one quadrant of the pool, 15 cm from the pool’s edge and submerged 1 cm beneath the water surface.

Hidden Platform Test: each rat was marked with yellow on the head and back with nitroxanthic acid, then trained in a circular pool which randomly divided into four equal quadrants by a hidden platform with four trials for 120 s per trial with a 30 min interval between the trials, daily, for five consecutive days. The time for the rat to seek the platform was recorded by an online image video tracking system and within 120 s as the escape latency.

Spatial Probe Test: the platform was removed from the pool after completion of the hidden platform test, each rat was left to one quadrant of the pool with a distance farthest from the primary platform. The number of times of the rat crossing the platform, the time spent in the target quadrant, and the running percentage of the target quadrant were recorded by the online image video tracking system.

### 2.6. Establishment of the Rat Model of AD

Male SD rats (*n* = 80) were selected by the Morris water maze according to the judgment standard of reference with the times to cross the platform between 40 and 120 s [37]. Then, the rats were randomly divided into a sham operation group (*n* = 12) and study group (*n* = 68). The rats in the study group were injected with 10 μg (2 μg/1 μL) state of aggregation Aβ_25–35_ in the lateral ventricle to establish the model of AD [38], while the rats in the sham operative group were injected with an equal volume of normal saline (NS) solution as control.

### 2.7. Drug Preparation and Administration

All treatments were initiated on day 21 after the operation. The rats with AD were randomly divided into five groups and treated with NS, donepezil 0.42 mg/kg (positive control), LSS 120, 240, and 480 mg/kg by intragastric (IG) administration once a day (daily) for 28 consecutive days. The rats in the sham operative group were given an equal volume of NS solutions by IG for 28 days as a negative control. The dose of donepezil was selected as previous described from the literature [39].

### 2.8. Histopathological Examination of Hippocampal Neurons of Rats

The rats were anesthetized by intraperitoneal (IP) injection of 1.0% pentobarbital, and perfusion of 4% paraformaldehyde to fix the brains for 24 h after NS or drug treatments. Then, the brains were removed and the brain tissues were weighed, fixed in 10% neutral buffered formalin, dehydrated, and embedded in paraffin for coronal microtome sections with H&E stain for the pathological study under light microscope (400×) as previously described [40].

### 2.9. Apoptosis Analysis of Neurons of Rats by Morphology and TUNEL

The apoptotic study was carried out with TUNEL analysis as previously described [41]. The same sections of neurons of rats for histopathological study were evaluated by morphology after H&E stain under microscope for distinct morphological features of apoptotic cells, including nuclear condensation and fragmentation. The presence of apoptotic cells was confirmed by TUNEL immunohistochemical assay on paraffin sections. The apoptotic cells showed as brown in color in the nuclei of cells. Apoptosis indices were calculated as the percentage of apoptotic cells among one hundred neuronal cells in a randomly selected portion. The positive rate of apoptotic cells was calculated by a GD-10.0 image analysis system.

### 2.10. RNA Isolation and Analysis of Caspase-3 mRNA Expression of the Brain Tissues of Rats by Reverse Transcription-Polymerase Chain Reaction

The brain tissues of rats were removed after anesthesia by IP injection of 1.0% pentobarbital. The total RNAs of the tissues were isolated and extracted by TRIzol followed to the general steps described from the literature [42,43]. Briefly, the tissues were washed with cold phosphate-buffered saline and cut into small pieces, 70 mg of tissue were homogenized in a mixture solution, 0.2 mL of chloroform was added after leaving at room temperature for 5 min, shocked for 15 s, and centrifuged at 12,000× *g* for 15 min at 4 °C, then transferred to a new vial and added isopropanol and precipitated for 10 min on the ice, centrifuged at 12,000× *g* for another 10 min at 4 °C then washing the precipitates with 75% alcohol and discarding the supernatants after being centrifuged at 7500× *g* for 5 min at 4 °C. The purity and concentration of RNAs were determined by Sepharose gel (Invitrogen, Carlsbad, CA, USA) and a UV spectrophotometer (Beckman-Coulter DU-640, Fullerton, CA, USA), respectively. The purity: A260/280 as 1.91–2.19 and A260/230 as 1.98–2.31; while the concentration > 0.2 µg/µL. One µg of RNA was conducted to reverse transcription to generate cDNA, the reaction of PCR was conducted through the primer sequences (Reaction conditions as shown in Table 1) according to the manufacturer’s protocol. The products of PCR were run on a 0.1% agarose gel electrophoresis with a DL500 DNA marker (Invitrogen, Carlsbad, CA, USA). Gel imaging and analysis were conducted for examination and quantitative analyses. β-actin was used as the load control.

### 2.11. Cell Culture

PC12 cells were purchased from the China Center for Type Culture Collection (CCTCC, Wuhan, Hubei, China) and cultured in Dulbecco’s modified Eagle’s medium (DMEM) supplemented with 5% fetal bovine serum, 10% horse serum, penicillin (100 U/mL), and streptomycin (100 μg/mL) in a CO_2_ incubator (5% CO_2_, 37 °C) and renewed with new medium every 3–5 days.

### 2.12. Western Blotting Analysis of the Protein Expressions of Bcl-2 and Bax in PC12 Cells In Vitro

First, PC12 cells were seeded at a density of 1.0 × 10^5^ cells/well on six-well plates (2.0 mL) and the cells were exposed to 20 μmol/L of Aβ_25–35_ in a CO_2_ incubator for 12 h to establish the cell model of AD. Then, the cells were randomly divided into five groups, rinsed with serum-free DMEM medium, and treated with the medium (control) or LSS (0.95, 1.90, 3.80, and 7.60 mg/L) together with 20 μmol/L of Aβ_25–35_ for 12 h (Aβ_25–35_ exposure to the cells for 24 h in total). The regular PC12 cells were also rinsed by serum-free DMEM medium and treated with the medium as an additional control.

After being cultured for 24 h, the culture media were discarded and the cells were washed with cold phosphate-buffered saline for harvest. The cell pellets were disrupted in cell RIPA buffer (0.5% NP-40, 50 mM Tris-HCl, 120 mM NaCl, 1 mM EDTA, 0.1 mM Na_3_VO_4_, 1 mM NaF, 1 mM PMSF, and 1 μg/mL leupeptin, pH 7.5) and collected after centrifuging at 800× *g* for 5 min at 4 °C, and the lysates were centrifuged at 9660× *g* for 15 min at 4 °C. The protein concentrations were determined by the phenylmethanesulfonyl fluoride (PMSF) method [44]. Equal amounts of proteins (30 μg) were electrophoresed on 7.5% density of sodium dodecyl sulfonate (SDS)-acrylamide gels. Following electrophoresis, the proteins were transferred from the gel to a nitrocellulose membrane using an electric transfer system. Non-specific binding was blocked with 5% skim milk in Tris-buffered saline with tween (TBST buffer, 5 mM Tris-HCl, 136 mM NaCl and 0.1% Tween-20, pH 7.6) for 1 h. The blots were incubated with antibodies against Bax (1:1000), Bcl-2 (1:1000) or β-actin (1:800) overnight at 4 °C and were washed three times with 1× TBST. Then, the blots were incubated for 1 h at room temperature with a 1:1000 dilution of horseradish peroxidase-labeled anti-rabbit or anti-mouse IgG and washed three times with 1× TBST. The membranes were developed by incubation within the ECL Western blotting detection reagents. Then, the protein expression levels of Bax and Bcl-2 were detected using the Quantity One System and the ratio of Bcl-2/Bax from the relative rates of Bax/β-actin and Bcl-2/β-actin were calculated. β-actin was used as the control.

### 2.13. Statistical Analysis

All data were expressed as mean ± standard deviation (SD). Statistical differences of the data among the means of two or more groups were analyzed using Student’s *t*-test and/or one-way univariate analysis of variance (ANOVA). A difference at *p* < 0.05 was considered to be statistically significant (marked as *). The higher significance level was set at *p* < 0.01 (marked as **).

## 3. Results

### 3.1. The Chemical Profile of LSS

**Figure 1:**
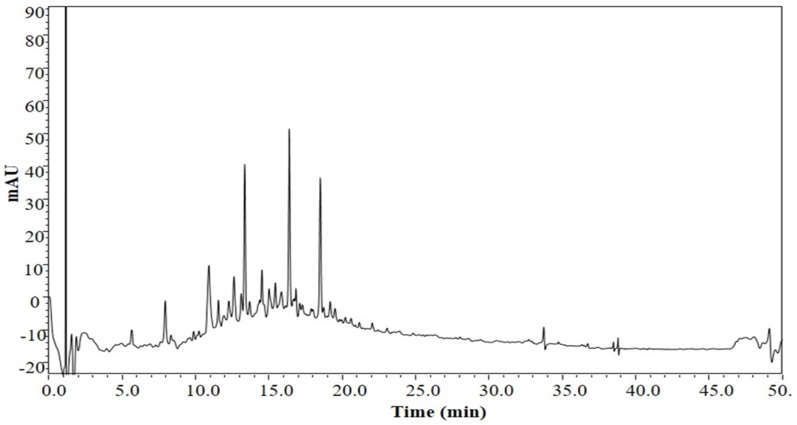
The chemical profile of LSS determined by a UHPLC-SPD chromatogram.

First, we isolated and extracted total saponins from lychee seeds. To determine the chemical profile of LSS, we studied the chemical fingerprint of LSS at 210 nm by a UHPLC-SPD chromatogram and the data are shown in Figure 1. The data reveal the main composition characteristic of LSS and indicate that it contains five major and 22 minor ingredients.

### 3.2. Validation of the Rat Model of AD by Escape Latency Test

In order to validate the AD model, the cognitive function of the rats was tested with escape latency in the normal rats (sham operative group, A) and the rats with AD (study group, B). The tested results were 10.18 ± 2.48 s in the sham operative (control) group and 50.94 ± 2.42 s in the study (model) group at the fifth day of positioning navigation training, respectively. The number was 80.01% by the formula of calculation of (B − A)/B%, suggesting the AD model induced by Aβ_25–35_ is validated according to the AD model criteria of (B − A)/B% > 20% [45].

### 3.3. Effect of LSS on the Ability of Spatial Learning and Memory of the Rats with AD

**Figure 2:**
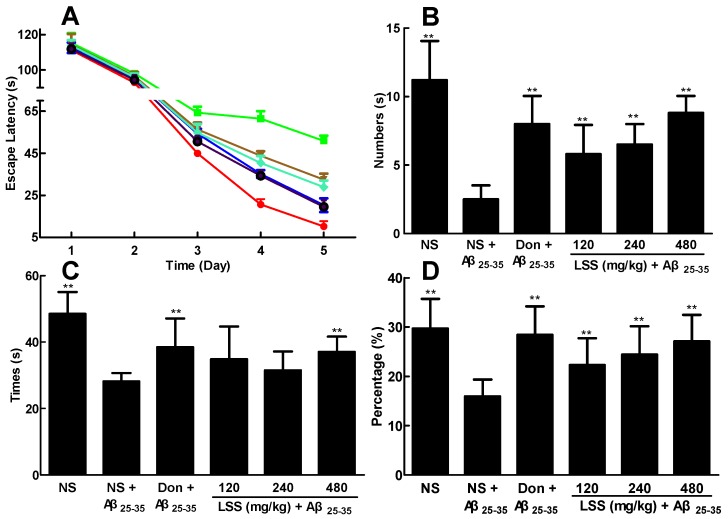
Effects of LSS and donepezil (Don) on the ability of spatial learning and memory in rats. (**A**). Escape latency: ● control rats treated with NS; ■ AD rats treated with NS; ▲ donepezil 0.42 mg/kg; ▼ LSS 120 mg/kg; ♦ LSS 240 mg/kg; 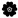 LSS 480 mg/kg; (**B**) Number of times across the platform; (**C**) platform quadrant dwell time; and (**D**) percentage of the total distance run in the platform quadrant. There were 10 rats used for each experimental group and expressed as the mean ± SD. ** *p* < 0.01 vs. the AD rats treated with NS (control).

After we established the rat model of AD, the ability of spatial learning and memory was investigated using the Morris water maze, a widely used method for assessing the cognitive process and a classic test for examining spatial learning and memory, in AD rats and compared to that of the sham operative rats (control). First, the hidden platform test was performed in the control rats and AD rats to investigate the ability of learning and memory of the rats. As shown in Figure 2A, the escape latency of the AD rats was markedly increased compared to that of the control rats (*p* < 0.01). However, LSS at the doses of 120, 240, and 480 mg/kg/day and donepezil at 0.42 mg/kg/day for 28 days by IG significantly shortened the escape latency in the AD rats compared to that of the AD rats treated with NS (*p* < 0.01).

Next, the spatial probe test was performed for the rats. Similarly, the numbers of platform crossings (Figure 2B), the time spent in the target quadrant (Figure 2C), and the run percentage in target quadrant (Figure 2D) were significantly shortened in the AD rats compared to those of the normal rats in the control group (*p* < 0.01). Once again, LSS (120, 240, and 480 mg/kg/day × 28 days, IG) significantly increased the numbers of rats crossing the platform (*p* < 0.01), the time spent in the target quadrant, and the run percentage in the target quadrant (*p* < 0.01) compared to that of NS treatment in AD rats. Donepezil (0.42 mg/kg/day × 28 days, IG) has the similarly effect as LSS at the dose of 480 mg/kg/day. These results suggest that LSS can improve the ability of spatial learning and memory of the AD rats.

### 3.4. Effect of LSS on the Neuroprotection in the Rats with AD

**Figure 3:**
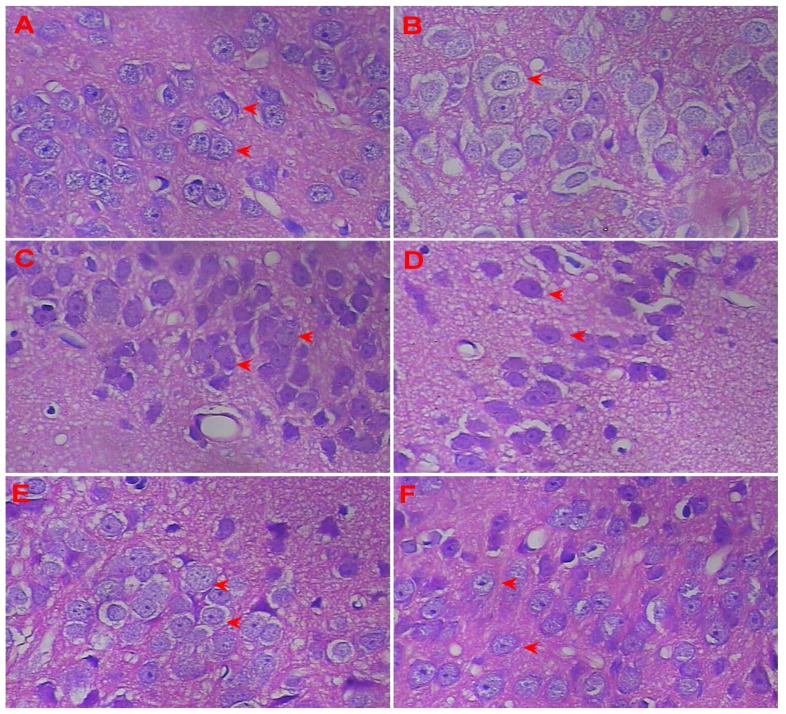
Protective effects of LSS and donepezil on neurons injury induced by Aβ_25–35_ in the hippocampus of AD rats (400×). (**A**) Histologic picture of neurons in the control rat treated with NS, shows preserved normal histological features of neurons; (**B**) Histologic picture of neurons in the AD rat treated with NS, shows damaged neurons; (**C**) Histologic picture of neurons in the AD rat treated with donepezil 0.42 mg/kg, shows near normal histological features of neurons; (**D**) Histologic picture of neurons in the AD rat treated with LSS 120 mg/kg, shows near normal histological features of neurons; (**E**) Histologic picture of neurons in the AD rat treated with LSS 240 mg/kg, shows near normal histological features of neurons; (**F**) Histologic picture of neurons in the AD rat treated with LSS 480 mg/kg, shows normal histological features of neurons. Each group had three rats.

The neurohistopathological changes were observed in the neuronal sections of the rat by H&E stain under a light microscope (400×) to examine the effect of LSS on neuroprotection. The representative morphologic pictures of neuronal cells of the rats are illustrated in Figure 3. The picture in Figure 3A shows the characteristic preserved normal histological features of the neuronal cells in the control rat (sham operative rat) treated with NS, in which the cells arranged closely and orderly, with a lightly stained large and round nucleus, clear nucleolus, no obvious signs of cytoplasmic or nuclear condensation, deep staining, and other neuronal degeneration and damage (red arrows in Figure 3A). However, the neuronal cells in the AD rat treated with NS (Figure 3B) display a loose arrangement with reduced numbers and spindle, irregular morphology, decreased cell volume from cytoplasm condensation, and karyopyknosis, deeply stained (red arrow in Figure 3B). Interestingly, the morphology of the cells is nearly normal, the cells arrange closely and orderly with increased cell numbers, with a large and round nucleus and clear nucleolus, in the groups of donepezil (0.42 mg/kg/day × 28 days, IG, red arrows in Figure 3C) or LSS (120, 240, and 480 mg/kg/day × 28 days, IG) in a dose-dependent manner (red arrows in Figure 3D–F).

### 3.5. Effect of LSS on Neuronal Apoptosis in the Rats with AD

**Figure 4:**
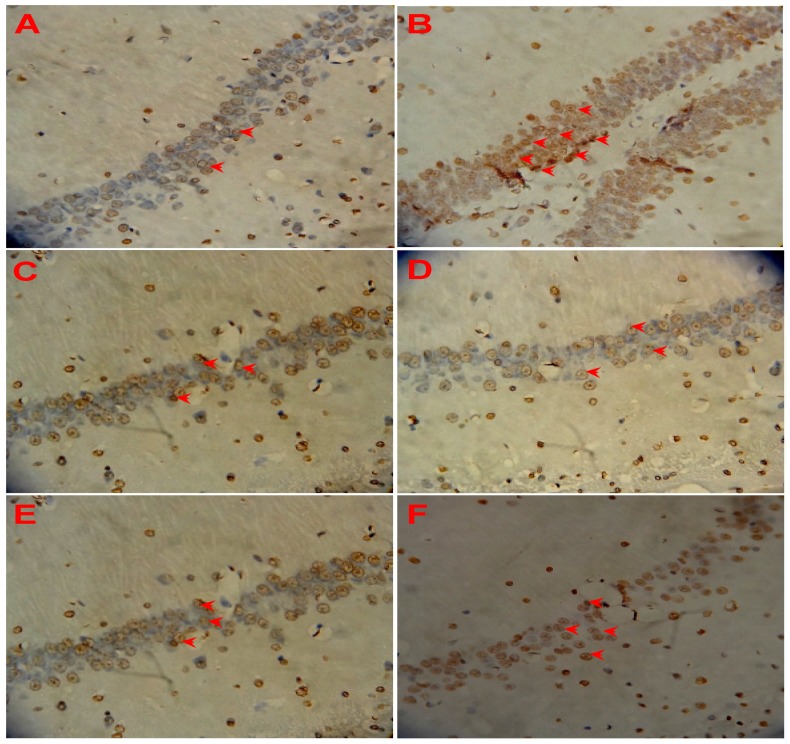
Effects of LSS and donepezil on neuronal apoptosis induced by Aβ_25–35_ in the hippocampus of control (normal) and AD rats (400×). (**A**) Neuronal cells in the control rat treated with NS; (**B**) Neuronal cells in the AD rat treated with NS; (**C**) Neuronal cells in the AD rat treated with donepezil 0.42 mg/kg; (**D**) Neuronal cells in the AD rat treated with LSS 120 mg/kg; (**E**) Neuronal cells in the AD rat treated with LSS 240 mg/kg; (**F**) Neuronal cells in the AD rat treated with LSS 480 mg/kg. The results are representative of at least three independent experiments.

**Figure 5:**
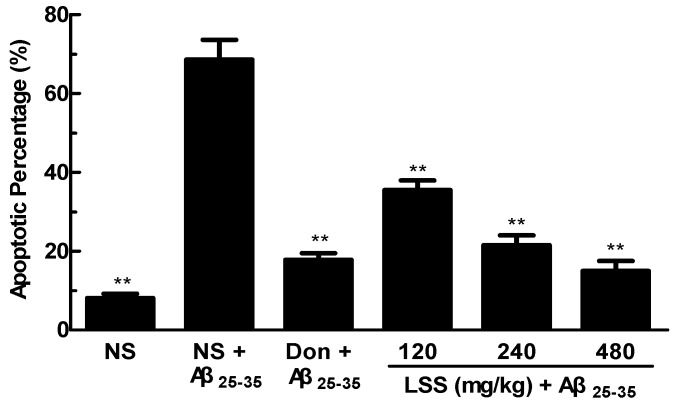
Percentages of apoptosis in the sections of the neuronal cells in the hippocampus of rats. The results are representative of at least three independent experiments and expressed as the mean ± SD. ** *p* < 0.01 vs. the AD rats treated with NS (control).

The crucial factor for the pathological changes and neuronal damage of AD is due to neuronal cell apoptosis. Therefore, we studied neuronal apoptosis in the sections of the control rats and AD rats by morphological and TUNEL immunohistochemical assays. The representative photographs are shown in Figure 4. We can see that a few spontaneous apoptotic cells are clearly observed which appear brown in the neurons of the control rat treated with NS, the spontaneous apoptotic cells are sparsely scattered in the viable portions of the neuronal tissues (Figure 4A). Higher levels of apoptosis are detected in the AD rats treated with NS (Figure 4B), and the apoptotic index of cells are obviously increased compared to that of the control rat (*p* < 0.01). However, the numbers of apoptotic cells are significantly decreased after the treatment of donepezil (0.42 mg/kg/day × 28 days, IG, Figure 4C) or LSS (120, 240, and 480 mg/kg/day × 28 days, IG, Figure 4D–F). The apoptotic index in the neuronal tissues is 8.10% ± 1.07% in the control rat (sham operative rats treated with NS); while the apoptotic index in the neuronal tissues is much higher than that of the control and reaching to 68.60% ± 5.02% in the AD rats treated with NS, however, the apoptotic index dropped to a level close to that of the control after the treatment of donepezil (17.80% ± 5.02%) or LSS (35.50% ± 2.43%, 21.50% ± 2.50%, and 15.00% ± 2.51% for 120, 240, and 480 mg/kg, respectively, Figure 5). The data indicate that neuronal apoptosis plays an important role in AD and LSS can effectively inhibit neuronal apoptosis.

### 3.6. Effect of LSS on the mRNA Expression of Caspase-3 in the Neuronal Tissues of the Rats with AD

**Figure 6:**
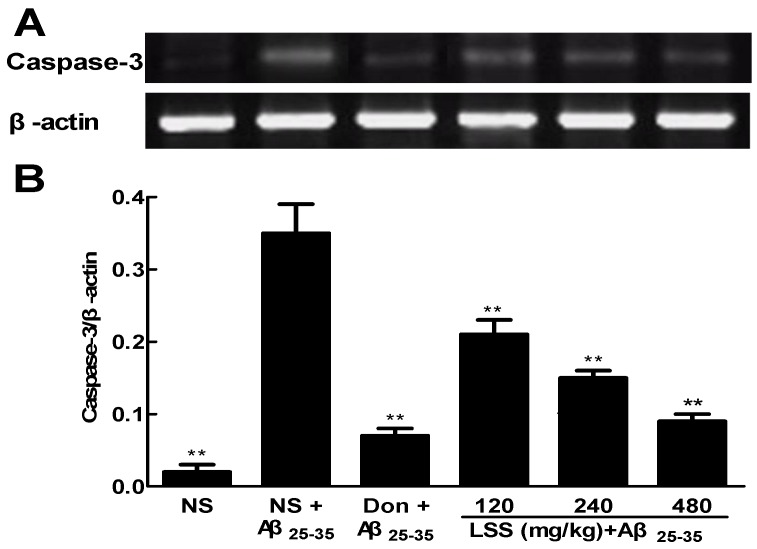
Effects of LSS and donepezil (Don) on the mRNA expressions of caspase-3 in the brain tissues of rats (**A**) The bands of caspase-3 mRNA and β-actin; (**B**) The ratio of caspase-3 mRNA and β-actin. The results are representative of at least three independent experiments and expressed as the mean ± SD. ** *p* < 0.01 vs. the AD rats treated with NS (control).

Caspase-3 is important or essential for certain processes of apoptosis. We speculated that the anti-apoptotic efficacy of LSS may be involved in the mRNA expressions of caspase-3 in the neuronal tissues in AD rats. Therefore, the expression levels of caspase-3 were analyzed by RT-PCR, and the intensities of bands of caspase-3 mRNAs and β-actin, as well as the relative ratio of caspase-3/β-actin are shown in Figure 6. The data show that the mRNA expression of caspase-3 increased significantly in the neuronal tissues in AD rats treated with NS compared to that of control rat treated with NS (*p* < 0.01). However, treatments of LSS (120–480 mg/kg) and donepezil (0.42 mg/kg) significantly decreased (*p* < 0.01) the elevated mRNA expression of caspase-3 in AD rats compared to that of NS treatment.

### 3.7. Effect of LSS on the Protein Expressions of Bax and Bcl-2 in the PC12 Cells Treated with Aβ_25–35_

**Figure 7:**
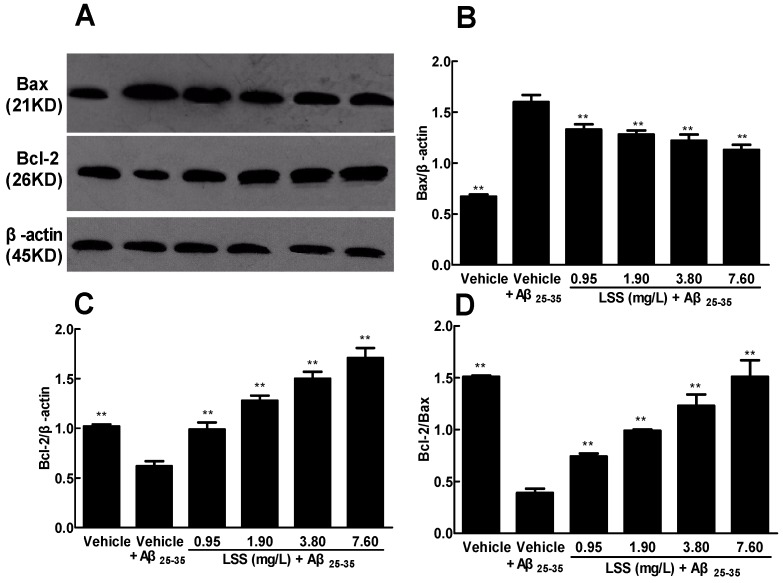
Effect of LSS on the protein expressions of Bax and Bcl-2 in PC12 cells. (**A**) The bands of Bax, Bcl-2 protein and β-actin; (**B**) The ratio of Bax protein and β-actin; (**C**) The ratio of Bcl-2 protein and β-actin; (**D**) The ratio of Bcl-2 protein and Bax. The results are representative of at least three independent experiments run in triplicate and expressed as the mean ± SD. ** *p* < 0.01, vs. the cells treated with Aβ_25–35_ and medium (vehicle control).

After we demonstrated that neuronal apoptosis played an important role in the pathogenesis of AD and the anti-AD efficacy of LSS in vivo, we further investigated the possible mechanism associated with the apoptotic pathway. We evaluated the protein expressions of Bax and Bcl-2 in the cultured PC12 cells treated with or without Aβ_25–35_ by Western blotting and the data are illustrated in Figure 7. The intensities of protein bands of Bax, Bcl-2, and β-actin in the cells with or without LSS treatment are shown in Figure 7A. The protein expression level of Bax is significantly upregulated (Figure 7A,B), while the protein expression level of Bcl-2 is significantly downregulated (Figure 7A,C) in the PC12 cells treated with Aβ_25–35_ compared to that of PC12 cells without Aβ_25–35_ (vehicle) treatment (*p* < 0.01). However, LSS at the concentrations of 0.95, 1.90, 3.80, and 7.60 mg/L for 12 h exposures significantly downregulated (*p* < 0.01) the expression level of Bax protein (Figure 7A,B) and upregulated (*p* < 0.01) the expression level of Bcl-2 protein (Figure 7A,C) compared to that of the PC12 cells treated with Aβ_25–35_ and medium (vehicle control). Furthermore, the relative ratio of Bcl-2/Bax is significantly decreased (*p* < 0.01) in the PC12 cells treated with Aβ_25–35_ compared to that of PC12 cells without Aβ_25–35_ (vehicle) treatment, however, LSS partially or completely revived the decrease induced by Aβ_25–35_ in a concentration-dependent manner (Figure 7D).These results indicate that the anti-AD effect of LSS may be associated with the protein expressions of Bax and Bcl-2 and it may be the possible mechanism for apoptosis inhibition.

## 4. Discussion

AD is a very common progressive neurodegenerative disorder and lacks an effective treatment. Currently, the drugs used for AD treatment can only relieve the symptoms of the patients, but cannot slow its progression or cure the disease [21]. In order to discover and develop more effective and less toxic novel anti-AD drugs or nutrient supplements, we isolated and extracted total saponins from lychee seeds and established a rat model of AD to evaluate the anti-AD efficacy of LSS and compared it to donepezil, the standard drug for AD treatment clinically. First, we analyzed the chemical profile of LSS and found five major and 22 minor ingredients (Figure 1). We demonstrated that LSS significantly improved the cognitive function in the AD rats for shortening the escape latency, increasing the number across the platform, platform quadrant dwell time, and the percentage of the total distance run platform quadrant compared to those of AD rats treated with NS (Figure 2). LSS also markedly alleviated the neuronal injury in the AD rats by morphological study (Figure 3).

In light of the recent investigation into the pathogenesis of AD, accumulating evidence from basic research in animal models of AD and clinical data has highlighted that the crucial factor for the neuronal injury and loss is due to neuronal apoptosis [5]. Apoptosis plays a critical role during the development of the nervous system and in many chronic neurodegenerative diseases including AD. Cells undergo apoptosis via two major pathways, the intrinsic (mitochondrial) pathway and extrinsic (death receptor-mediated) pathway. The intrinsic apoptotic process occurs in the mitochondria and mainly affects the Bcl-2 family and caspases. The extrinsic pathway of apoptosis involves the interaction of death signals (such as TNF-α with TNFR1) and activates caspase-8 to cleave procaspase-3 to its active form [45]. The Bcl-2 family consists of pro-apoptotic proteins (Bax, Bad, and Bak) and anti-apoptotic proteins (Bcl-2 and Bcl-xL). BAD can increase mitochondrial permeability, release pro-apoptosis factors and CytC, trigger caspase signaling through inhibiting anti-apoptosis Bcl-2 family proteins, resulting in neuronal apoptosis [20,46].

The ratio of pro-apoptosis proteins to anti-apoptosis proteins directly determines the open degree of various channels in the mitochondrial outer membrane of cells and regulation of apoptosis [47]. Bcl-2 and Bax proteins play a crucial role in signal transduction in apoptosis. Caspase-3 is an important regulator of apoptosis and its activation has been identified as a key step in the process of cell apoptosis [48,49]. Activated caspases cleave a variety of target proteins [50], resulting in the breakdown of DNA fragments in the cell [51], and finally lead to cell apoptosis [52]. In addition, caspase-3 also plays a crucial role in the ultimate apoptosis of cells in AD with tightly linked to the proteolytic cleavage of polymeric tau protein [53] and formation of Aβ peptides [54].

**Figure 8:**
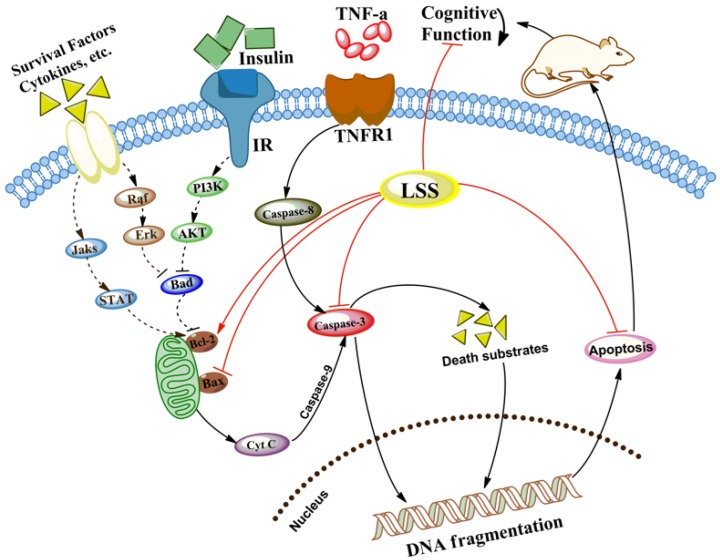
LSS improves cognitive function and prevents neuronal injury of AD rats via inhibiting the neuronal apoptosis signaling pathway.

The present studies with the rat model of AD induced by Aβ_25–35_ demonstrated that neuronal apoptosis plays a key role in the pathogenesis of AD, and LSS can significantly inhibit neuronal apoptosis (Figure 3 and Figure 4). Furthermore, we also analyzed the mRNA expression of caspase-3 in the neuronal tissues of rats and the protein expressions of Bax and Bcl-2 in PC12 cells, our results show that Aβ_25–35_ significantly increased the expression of caspase-3 in the brain tissues of the AD rats compared to that of the control rats, while LSS significantly reduced the expressions of caspase-3 mRNA and Bax protein, meantime, increased the protein expression of Bcl-2 and the ratio of Bcl-2/Bax. Those results suggest that LSS can improve cognitive function and prevent neuronal injury in AD rats via inhibiting neuronal apoptosis signaling pathway (Figure 8).

Yang and Liang reported anti-diabetic effect of LSS and determined its active contents [36]. We also analyzed the chemical profile of LSS and found that it contains five major and 22 minor ingredients (Figure 1). We have further separated the ingredients systematically by preparative HPLC. However, we still have a problem in terms of structural identification by high-resolution mass spectrum ^1^H-NMR or ^13^C-NMR due to the purity and quantity of LSS. Therefore, we have sent the samples to a professional analysis company for structural identification and other studies. Our previous studies have demonstrated that lychee seed extracts can improve IR in the animal models of Type II diabetes mellitus with AD-like cognitive impairment (unpublished data), suggesting that the protective effect of LSS on neuronal injury and loss may be associated with the insulin signaling pathway by restoring the weakened signaling caused by Aβ. However, it is unclear whether the protective effect of LSS on the neurons of rats is due to regulation of apoptotic pathway directly or via improvement of IR to further after cell apoptosis. It needs to be further investigated. However, the effects of LSS on the expressions of caspase-3, Bax, and Bcl-2 suggest that the anti-AD effect of LSS may also be related to mitochondrial permeability, CytC, and PI_3_K/Akt signal pathway. Therefore, further studies are needed to determine the mechanistic action of LSS on the associated molecular pathways of neuronal apoptosis in neurodegeneration of AD.

## 5. Conclusions

We have demonstrated that LSS contains five major and 22 minor ingredients and it significantly improves cognitive function and obviously prevents neuronal injury induced by Aβ_25–35_ in AD rats through inhibiting apoptosis of the neuronal cells in the hippocampus. LSS is similarly, or even more, effective against AD than donepezil (a classic drug for AD treatment clinically) in the model system. Furthermore, LSS can downregulate the expressions of caspase-3 mRNA and Bax protein and, at the same time, upregulate the expression of Bcl-2 protein in a concentration-dependent manner. Therefore, LSS has the potential to be developed as a novel drug or nutrient supplement for the prevention and/or treatment of AD clinically.

## Figures and Tables

**Table 1:** The primer sequence, PCR target, and cycle for mRNA of caspase-3 and β-actin.

mRNA	Primer Sequence	PCR Target (bp)	Cycle
Caspase-3	F: 5′-GAACGATCGGACCTGTGG-3′ R: 5′-GGGTGCGGTAGAGTAAGC-3′	218	30
β-actin	F: 5′-CTGGAAGGTGGACAGTGAG-3′ R: 5′-GAGGGAAATCGTGCGTGAC-3′	445	30
